# Competitive Interactions Among *Populus euphratica* Seedlings Intensify Under Drought and Salt Stresses

**DOI:** 10.3390/plants14243842

**Published:** 2025-12-17

**Authors:** Xiao-Hui Li, Xue-Ni Zhang, Shuang-Fu Zhou, Hui-Xia Li, Yu-Fei Chen

**Affiliations:** 1 College of Ecology and Environment, Xinjiang University, Urumqi 830017, China; 107552301778@stu.xju.edu.cn (X.-H.L.); zshuangfu000@163.com (S.-F.Z.); lihuixia07@stu.xju.edu.cn (H.-X.L.); chenyufei@stu.xju.edu.cn (Y.-F.C.); 2 Key Laboratory of Oasis Ecology, Ministry of Education, Xinjiang University, Urumqi 830017, China; 3 Xinjiang Jinghe Observation and Research Station of Temperate Desert Ecosystem, Ministry of Education, Jinghe 833300, China

**Keywords:** plant interactions, intraspecific competition, drought and salt stress, desert plants, SGH

## Abstract

Plant interactions and their responses to stress environments are important ecological processes for ecosystem stability and biodiversity formation, but how plant intraspecific relationships respond to environmental stresses remains to be studied in depth. In this study, annual *Populus euphratica* seedlings were planted in singles or doubles, and two stress treatments were set up: two drought levels (0.7 and 0.4 L) and two salinity levels (200 and 400 mmol L^−1^). *P. euphratica* seedlings’ total and part biomass, root/shoot ratio, net photosynthetic rate, stomatal conductance, nonstructural carbohydrate concentration, and proline content were measured. Relative interaction indices were calculated to clarify their intraspecific relationships. The results of the study showed that compared to the single-planted *P. euphratica*, the double-planted *P. euphratica* was more significantly inhibited by drought and salt stress, the total biomass decreased, photosynthesis declined, proline content increased, and non-structural carbohydrates changed, which reflected a competitive intraspecific relationship. Secondly, as drought and salt stress intensified, the relative interaction index indicated that the intraspecific relationship of *P. euphratica* seedlings gradually shifted from neutrality to competition, which indicated that the intraspecific competitive relationship of *P. euphratica* seedlings was exacerbated by environmental stresses. These findings highlight the need to account for stress-mediated competition in *P. euphratica* seedlings during ecological restoration in arid environments.

## 1. Introduction

Plant interactions and their responses to stress environment play an important role in species distribution, biodiversity and their coexistence mechanisms, and are one of the topical issues of interest in community ecology [1,2]. Intraspecific relationships in plants refer to the interaction of individuals or populations of organisms of the same species with each other in the process of survival, mainly including intraspecific competition and intraspecific promotion [2,3]. In-depth study of intraspecific relationships and their environmental response issues can more accurately predict the response of different plant survival conditions and distribution to environmental stresses such as climate change, and help to develop more effective management and conservation strategies [4]. In arid ecosystems, desert plants chronically endure abiotic stressors such as drought and salinity, resulting in complex adaptive traits in their intraspecific relationships [5]. Consequently, examining the interactions between plant functional traits and environmental factors is critical for elucidating the driving mechanisms and influencing factors of intraspecific relationships, while providing a scientific basis for biodiversity conservation practices [6].

Plant interactions have received growing attention in ecology, as they play an important role in shaping community structure and function, especially in resource-limited environments [7]. For example, some plant species can distinguish neighboring plants and may adjust their own root growth accordingly, thereby changing the relative strength of interspecific competition and potentially modifying theoretical predictions of species coexistence [8]. However, while interspecific interactions have been extensively studied, intraspecific relationships—particularly in extreme environments like deserts—have received comparatively less attention. This gap limits our mechanistic understanding of how plants interact within species under arid conditions [9]. Stress gradient hypothesis (SGH) is a traditional theoretical framework of plant interactions, which suggests that plant interactions change from competition to facilitation as the level of environmental stress increases [10,11]. The traditional SGH was initially widely supported, but expanded research came to divergent conclusions. Maestre et al. refined the hypothesis by integrating plant life history traits with stressor characteristics, proposing that the outcome of plant interactions along stress gradients depends on the specific combination of these factors [12]. This refined SGH better explains interspecific relationships, including variations under different abiotic stresses like those in arid regions [9,11,13,14]. However, whether intraspecific relationships among early seedling stages of woody plants in arid regions, such as *Populus euphratica*, conform to the predictions of the refined SGH, particularly across distinct stress gradients like drought and salinity, remains insufficiently supported by empirical evidence [4,12].

Intraspecific interactions in plants play a critical role in regulating growth, reproductive success, and developmental processes. These interactions engage in complex interplay with plant stress responses and environmental factors [4,15,16,17]. As sessile organisms, plants have evolved adaptive strategies centered on resource allocation and cellular osmotic adjustment to cope with environmental stresses—responses that directly influence the intensity of intraspecific competition [18,19]. Under drought and salinity stress, plants optimize root architecture to enhance water acquisition while reducing stomatal conductance to minimize water loss; this dual regulatory mechanism inevitably constrains photosynthetic rates [20,21]. Plants also maintain cellular osmotic balance by altering the composition and concentration of non-structural carbohydrates, typically increasing soluble sugars while decreasing starch levels [22,23]. Furthermore, accumulation of osmoprotectants such as proline enhances stress resilience [24]. These stress response mechanisms modulate the intensity of intraspecific interactions by altering resource acquisition and utilization efficiency. For instance, under saline-alkaline stress, plants employ specific adaptive strategies such as osmotic adjustment and ion exclusion to mitigate stress, thereby influencing reproductive output and mortality, which ultimately reshapes competitive relationships [25,26]. Conversely, intraspecific interactions can feedback onto plant stress responses: in high-density populations, competition for limited water and nutrients exacerbates drought stress effects [27,28]. However, the specific mechanistic basis underlying the interactions among environmental factors, intraspecific relationships, and stress responses—and how these effects vary across species and environments—remains poorly understood [29].

As a foundational species in the desert ecosystem of northwest China, *P*. *euphratica* exhibiting exceptional drought tolerance, salinity-alkalinity resilience, and adaptability to nutrient-poor soils. These characteristics make it a valuable resource for arid regions restoration and sustainable utilization [30]. Research indicates that *P. euphratica*, through its growth and distribution, may stabilize desert ecosystems and enhance biodiversity, fulfilling key ecological roles. Drought and salinization are recognized as critical abiotic stressors affecting interrelationships among desert flora and community distributions. Recent studies have highlighted that global climate change is likely to result in prolonged and intensified droughts, exacerbating issues such as water scarcity and soil salinization in the arid regions of northwest China [31,32]. In light of these challenges, it is hypothesized that adaptive mechanisms in *Populus euphratica* could lead to intraspecific competitive dynamics; however, empirical evidence supporting this conjecture remains insufficient [13].

*Populus euphratica* has emerged as an ideal model plant for investigating the complex relations between intraspecific relationships and plant stress adaptation in arid regions [30,33]. Therefore, our work was to study the differences in growth and morphological characteristics, photosynthetic physiological traits, non-structural carbohydrates, and proline accumulation characteristics between single-planted and double-planted *P. euphratica* seedlings and their stress responses, based on which we analyzed the intraspecific relationships of *P. euphratica* seedlings and their response patterns to different drought and salinity stresses. We hypothesized that (1) compared to single-planted *P. euphratica*, double-planted *P. euphratica* seedlings would exhibit more negative effects under drought and salt stress, reflecting intensified intraspecific competition. (2) Intensifying drought and salt stress would amplify this intraspecific competition between *P. euphratica* seedlings, and the competitive intensity might differ between these two stress types based on the refined SGH framework.

## 2. Results

### 2.1. Effects of Stress on Morphological Features

**Figure 1:**
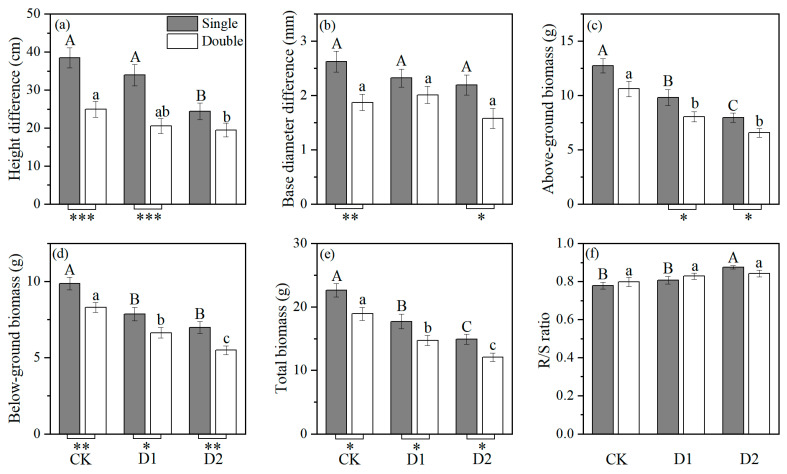
Effects of drought stress (CK, D1, D2) and plant number (single or double) on *P. euphratica* seedlings: (**a**) height difference, (**b**) basal diameter difference, (**c**) above-ground biomass, (**d**) below-ground biomass, (**e**) total biomass, and (**f**) root/shoot ratio. Different letters (A, B, C or a, b, c) indicate statistically significant differences between the three treatment levels for single or double-planted *P. euphratica* seedlings (*p* < 0.05). * Indicate statistically significant differences between single and double planted *P. euphratica* in each treatment (* *p* < 0.05; ** *p* < 0.01 and *** *p* < 0.001). Bars are means values (±s.e., *n* = 6).

Under control (CK) and drought stress treatments (D1, D2), height difference, basal diameter difference, and biomass were higher in single *Populus euphratica* than in double and were significantly different in most of the cases, which suggests that the growth of double-planted *P. euphratica* was more significantly inhibited compared to that of *P. euphratica* grown alone (Figure 1). In addition, compared with CK, drought stress had a significant negative effect on all morphological characters, particularly reducing above-ground, below-ground and total biomass (Figure 1; Table 1).

**Figure 2:**
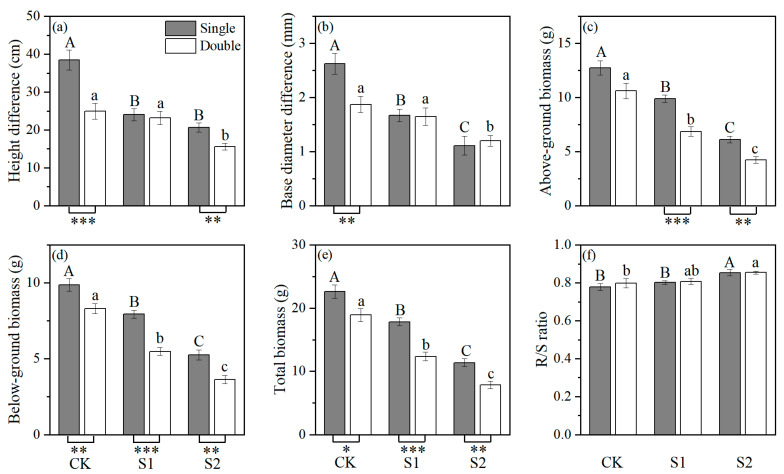
Effects of salt stress (CK, S1, S2) and plant number (single or double) on *P. euphratica* seedlings: (**a**) height difference, (**b**) basal diameter difference, (**c**) above-ground biomass, (**d**) below-ground biomass, (**e**) total biomass, and (**f**) root/shoot ratio. Bars are means values (±s.e., *n* = 6). The statistical analysis is shown in Figure 1.

In salt stress (S1, S2), the biomass of double-planted *P. euphratica* was significantly lower than that of single-planted *P. euphratica*, indicating that the growth of double-planted *P. euphratica* was more significantly inhibited. In addition, the height difference of the double-planted was significantly lower than that of the single-planted at CK and S2 (Figure 2). Salinity stress had a significant effect on all morphological characters, and plant number had a significant effect on height difference and biomass (Table 1).

### 2.2. Effects of Stress on Photosynthetic Physiology

**Figure 3:**
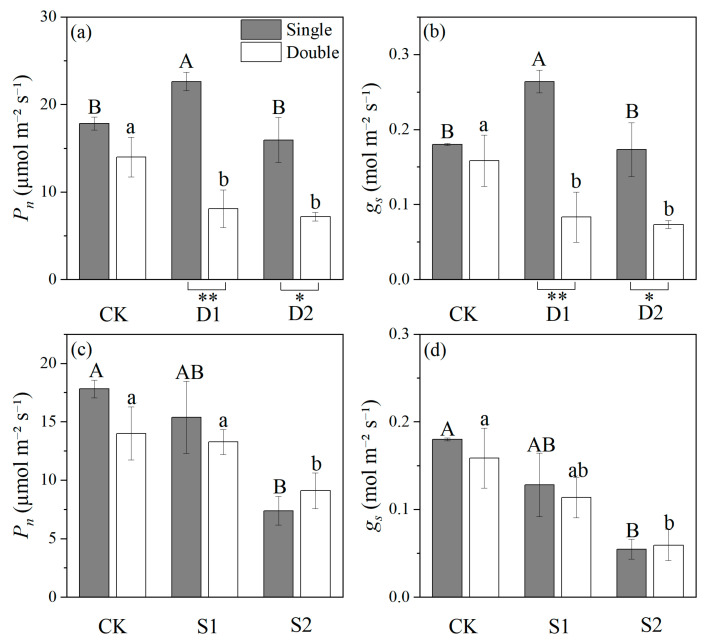
Effects of drought stress (CK, D1, D2) and plant number (single or double) on *P. euphratica* seedlings: (**a**) net photosynthetic rate (*P_n_*) and (**b**) stomatal conductance (*g_s_*). Effects of salt stress (CK, S1, S2) and plant number (single or double) on *P. euphratica* seedlings: (**c**) net photosynthetic rate (*P_n_*) and (**d**) stomatal conductance (*g_s_*). Bars are means values (±s.e., *n* = 6). The statistical analysis is shown in Figure 1.

Plant number had no significant effect on *P_n_* and *g_s_* in CK, but *P_n_* and *g_s_* were significantly higher in single-planted *P. euphratica* than in double-planted under both drought stresses. In addition, under drought stress, *P_n_* and *g_s_* of single-planted *P. euphratica* showed a tendency of increasing and then decreasing, and D1 was significantly higher than the other two groups, while the double-planted *P. euphratica* showed a decreasing trend, CK was significantly higher than the stress group (Figure 3).

The plant number did not significantly affect *P_n_* and *g_s_* across the salt stress treatments, but the single-planted pattern showed slightly higher *P_n_* and *g_s_* than the double-planted pattern under CK and S1 conditions, while an opposite trend in *P_n_* was observed under S2. Compared with the CK, the stress conditions showed a significant decrease in *P_n_* and *g_s_* of *P. euphratica* seedlings (Figure 3). In summary, salt stress and plant number had significant effects on both *P_n_* and *g_s_* (Table 1).

### 2.3. Effect of Stress on Chemical Features

**Figure 4:**
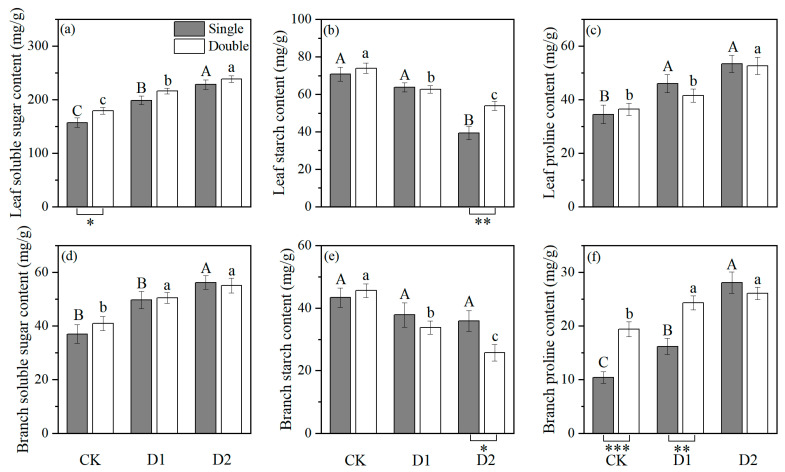
Effects of drought stress (CK, D1, D2) and plant number (single or double) on *P. euphratica* seedlings: (**a**) leaf soluble sugar content, (**b**) leaf starch content, (**c**) leaf proline content, (**d**) branch soluble sugar content, (**e**) branch starch content, and (**f**) branch proline content. Bars are means values (±s.e., *n* = 6). The statistical analysis is shown in Figure 1.

Under drought stress treatment, there were differences in starch content between single-planted and double-planted *P. euphratica* in the D2 treatment; and in CK and D1, the proline content of double-planted *P. euphratica* was significantly higher than that of single-planted. Compared with CK, under drought stress, the soluble sugar content of leaves increased significantly, but the starch content decreased, and the proline content of branches and leaves increased (Figure 4). In addition, drought stress had a significant effect on the soluble sugar, starch, and proline content of poplar leaves, and the number of plants had a significant effect on the starch content of leaves and branches (Table 1).

**Figure 5:**
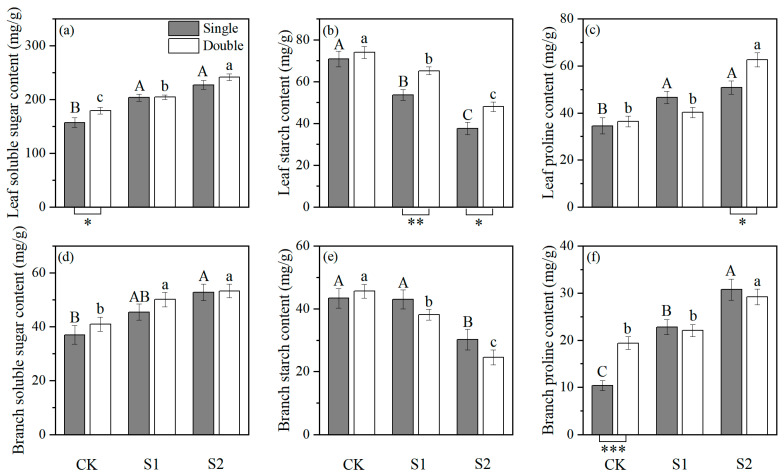
Effects of salt stress (CK, S1, S2) and plant number (single or double) on *P. euphratica* seedlings: (**a**) leaf soluble sugar content, (**b**) leaf starch content, (**c**) leaf proline content, (**d**) branch soluble sugar content, (**e**) branch starch content, and (**f**) branch proline content. Bars are means values (±s.e., *n* = 6). The statistical analysis is shown in Figure 1.

Under salt stress treatment, leaf starch content of single-planted *P. euphratica* was significantly lower than that of double-planted. As salt stress intensified, the soluble sugar and proline content of leaves and branches increased significantly, while the starch content decreased significantly (Figure 5). Salinity stress had significant effects on most of the chemical characteristics (Table 1).

### 2.4. Intraspecific Relationships of P. euphratica Seedlings and Their Changes Across the Stress Gradient

**Figure 6:**
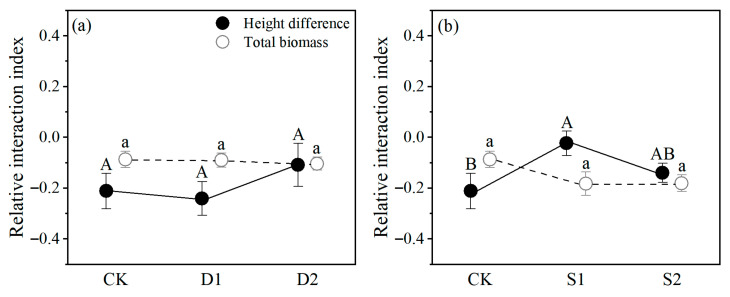
Relative interaction index (RII) of height difference and total biomass of *P. euphratica* during (**a**) drought stress and (**b**) salt stress. Different letters (A, B, C or a, b, c) indicate statistically significant differences in height difference or total biomass at *p* < 0.05. Circles are means values (±s.e., *n* = 6).

In the drought stress, the RII values of height difference and total biomass of *P. euphratica* seedlings were negative, reflecting a competitive intraspecific relationship. The RII value of height difference increased at D2 compared with CK and D1, while the average RII value of total biomass stabilized at −0.1. This indicates that as stress intensified, the intraspecific competitive relationship among *P. euphratica* seedlings does not show significant changes and gradually tends toward neutrality (Figure 6a).

In the salt stress, the RII values for height difference and total biomass were generally negative, reflecting a competitive relationship between *P. euphratica* seedlings. The RII values of height difference reflected a trend of increasing and then decreasing with stress intensified, and S1 was significantly higher than CK. There was no significant difference in the RII values of total biomass, but the RII values of CK were slightly higher, suggesting that salt stress exacerbated intraspecific competition in *P. euphratica* seedlings (Figure 6b).

**Figure 7:**
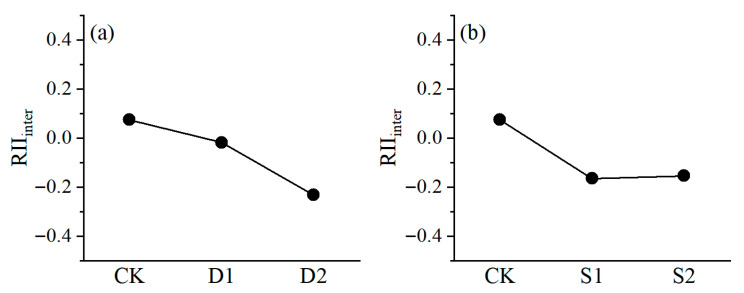
RII_inter_ of *P. euphratica* seedlings obtained by PCA under (**a**) drought stress and (**b**) salt stress. Circles are mean values (±s.e., *n* = 6).

The first three principal components were selected for multivariate statistics from the loading matrix and variance interpretation of the principal component analysis to derive the RII_inter_. The RII_inter_ value of CK is 0.07, reflecting a neutral effect. Under drought stress treatment, RII_inter_ value gradually decreases as the stress intensity increases, indicating that the intraspecific relationship of *P. euphratica* seedlings changes from neutral to competitive (Figure 7a). Under salt stress treatment, the RII_inter_ value decreased from 0.07 to −0.16, and the RII_inter_ values of S1 and S2 did not change significantly, but still reflected a competitive relationship. These results indicate that as stress levels increase, intraspecific relationships between *P. euphratica* seedlings shift from neutral to competitive (Figure 7b).

## 3. Discussion

### 3.1. Morphological and Physiological Characteristics of Double-Planted P. euphratica Seedlings Responded More Significantly to Drought Stress

In order to better adapt to intraspecific competition and stressful environments, *P. euphratica* exhibits some plasticity, such as improving its competitiveness by altering root structure, adjusting biomass allocation and photosynthetic activity [34,35]. Experiments have indicated that drought stress significantly impairs the growth of *P. euphratica* seedlings, consistent with previous studies [30,36]. Experimental data showed that under drought stress, the net photosynthetic rate and stomatal conductance of double-planted *P. euphratica* were significantly lower than those of single-planted *P. euphratica*, indicating that double-planted *P. euphratica* was subjected to competition-induced inhibition (Figure 3), which is similar to the conclusion of the study that intraspecific competition in potato plants affects their photosynthetic efficiency [37].

Compared with the control group, low drought stress significantly reduced net photosynthesis and stomatal conductance in double-planted *P. euphratica*, while slightly increasing them in single-planted *P. euphratica* (Figure 3a,b). It has been suggested that this is an adaptive response of plants to mild water stress, possibly due to the accumulation of solutes such as proline, which reduces the osmotic potential of cells [38]. This is consistent with the results of this study (Figure 4c). Moreover, plants’ enhanced ability to improve water use efficiency (WUE) and enhance reactive oxygen species (ROS) clearance orchestrates their adaptation to stress [36,38].

Some studies have suggested that nonstructural carbohydrates in plants, especially soluble sugar content, are critical for plant survival and stress tolerance and are closely related to physiological regulation [23,39]. The results of this experiment showed that drought stress increased the soluble sugar content and decreased the starch content of leaves and branches of *P. euphratica* (Figure 4), which was consistent with previous studies [23]. The soluble sugar content of double-planted *P. euphratica* is slightly higher than that of single-planted *P. euphratica*, which means that the former may have greater osmotic adjustment ability when subjected to drought stress. In addition, the drought stress × plant number interaction had a significant effect on the leaf starch content of the plants (Table 1), which is a good indication that the adaptation of *P. euphratica* seedlings to drought environments is affected by intraspecific relationships at the same time. Overall, under the combined effects of plant number treatment and drought stress, the growth morphology and physiological responses of double-planted *P. euphratica* were more severely impaired than those of single-planted *P. euphratica*. In other words, under drought stress environment, intraspecific relationship of *P. euphratica* seedlings generally reflects competition.

### 3.2. Morphological and Physiological Traits of Double-Planted P. euphratica Seedlings Responded Significantly Under Salt Stress

In this study, salt stress significantly reduced morphological traits such as height and biomass of *P. euphratica* seedlings, especially for the double-planted *P. euphratica* (Figure 2), suggesting competitive intraspecific relationship between *P. euphratica* seedlings. In addition, salt stress significantly reduced the net photosynthetic rate and stomatal conductance of *P. euphratica* seedlings (Figure 3c,d). This is because salt stress damages the function and structure of plant leaves, leading to a decrease in photosynthetic physiological traits [33]. We found that under low salinity stress, the photosynthetic traits of double-planted *P. euphratica* were lower than those of single-planted *P. euphratica*. However, under high salinity stress, single-planted *P. euphratica* exhibited a greater decline in photosynthetic traits and were more severely affected than double-planted *P. euphratica*. This shift implies a transition in intraspecific relationships from competition to facilitation under high salinity, aligning with the SGH. The observed facilitation may stem from root interactions that ameliorate rhizosphere conditions—such as enhanced water retention or collective alleviation of ionic stress—thus buffering the photosynthetic apparatus against severe salt damage [10,40]. Similar root-mediated facilitation has been documented in other woody species facing osmotic stress, supporting this interpretation [41]. Environmental stressors and plant intraspecific relationships jointly regulate physiological responses, although their relative importance may vary across stress regimes [42].

Among the nonstructural carbohydrates, starch serves as a long-term carbon store, providing energy for plants. Under salt stress, the leaf starch content of double-planted *P. euphratica* was significantly higher than that of single-planted individuals (Figure 5b). This higher starch accumulation likely represents a key physiological response to stress, enhancing carbon availability to fuel essential survival mechanisms under challenging conditions [23,39]. High salinity stress caused a significant increase in proline content of double-planted *P. euphratica* leaves, indicating that the *P. euphratica* may be undergoing osmotic adjustment to relieve extreme salinity stress and intraspecific competition [24]. Under high salinity stress, proline content in *P. euphratica* leaves and branches increased by an average of 60% and 100% relative to the control, respectively, indicating that proline accumulation may enhances salt tolerance capacity of *P. euphratica* [33]. In addition, the salt stress × plant number interaction had a significant effect on leaf proline content (Table 1), suggesting that plants accumulate large amounts of proline to sustain their own growth under the combined influence of salt stress and intraspecific relationships. Combining all the traits of single-planted and double-planted *P. euphratica* seedlings under salt stress, we concluded that the morphological characteristics and physiological responses of double-planted *P. euphratica* were more affected by salt stress, suggesting that intraspecific relationships of *P. euphratica* seedlings under salt stress is competitive.

### 3.3. Intraspecific Competition in P. euphratica Increases with Stress Increase

In this study, we first calculated RII based on the height difference and total biomass of *P. euphratica* seedlings to evaluate the intraspecific relationships and their changes under stress (Figure 6). We found negative RII values for both height difference and biomass under drought stress, reflecting a competitive relationship, consistent with our expectations and previous findings [14,27]. Under salt stress, the RII value for height difference exhibited a unimodal curve below 0, likely attributed to stress-sharing between paired *P. euphratica* seedlings, which mitigated intraspecific competition [33]. Overall, as stress increased, the changes in RII values based on the height difference and total biomass of *P. euphratica* seedlings varied slightly, but always indicated intraspecific competition, and competition intensified as stress increased.

Given that plant traits interact synergistically, we quantified RII_inter_ using the “integration” index derived from principal component analysis (Figure 7). The RII_inter_ value of the control group is positive, which indicates a neutral-promoting intraspecific relationship among *P. euphratica* seedlings under normal conditions. As drought stress intensifies, the RII_inter_ value continues to decrease, and intraspecific competition intensifies under high drought stress. This phenomenon likely arises because under extreme water scarcity, competition for limited water resources between individual in double-planted *P. euphratica* seedlings intensifies, thereby enhancing intraspecific competition [43]. Under salt stress treatment, the RII_inter_ value shifted from neutral in the control group to competitive under both salinity levels. This shift may be attributed to two main factors: first, the species’ inherent salt tolerance threshold necessitates increased resource allocation to stress defense under elevated salinity, intensifying competition; second, it results from the combined effects of seedling stress adaptation and neighbor interactions in double-planted conditions [15,44]. Furthermore, the comparable RII_inter_ values between the two salinity levels suggest consistently strong competitive intensity across the salt stress gradient. This observed shift from neutrality to competition with increasing stress aligns with refinements to the classical SGH. As introduced earlier, the SGH has been refined to account for such context-dependent shifts, including transitions to competition under severe resource limitation [28,45,46]. Our findings in *P. euphratica* seedlings thus provide a significant case of intraspecific interactions that not only aligns with but also empirically supports these refined SGH models—highlighting a key departure from the traditional hypothesis by demonstrating competition intensification under abiotic stress.

**Figure 8:**
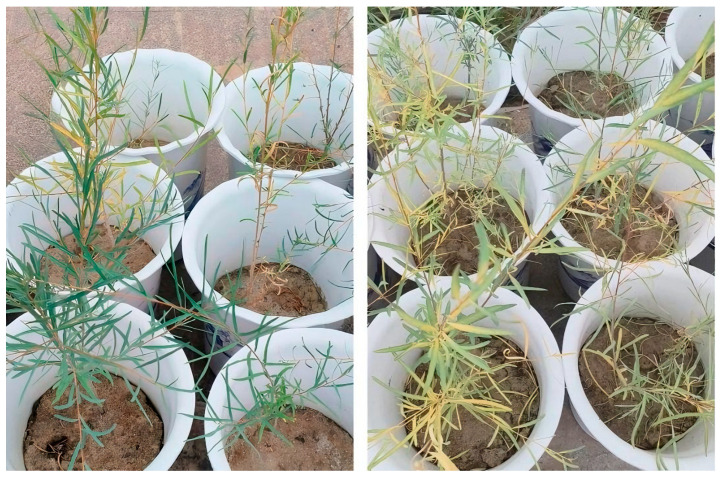
Performance of single-planted (left) and double-planted (right) *P. euphratica* seedlings under high drought stress (D2).

This study indicates that *P. euphratica* seedlings exhibit competitive intraspecific relationships under drought and salinity stress. Beyond influencing individual seedlings, this stress-mediated intensification of competition has implications for community assembly [47]. In arid ecosystems like those dominated by *P. euphratica*, strong abiotic filtering combined with intensified intraspecific competition can act as coexisting filters that shape population structure and potentially reduce intraspecific functional diversity [47,48,49]. The practical manifestations of these competitive relationships were visually evident in our experiment. In the later stage of stress in this experiment, leaves of some *P. euphratica* seedlings showed yellowing, wilting or even shedding, especially under high drought and salinity stress, and these phenomena resulted in a larger value of the root/shoot ratio of the *P. euphratica* seedlings. It is worth noting that these phenomena were mostly found in double-planted *P. euphratica*. For example, under high drought stress treatment, all six pots of double-planted *P. euphratica* had yellowing of leaves, while only three pots of single-planted *P. euphratica* were found, and it was evident that the growth performance of the double-planted *P. euphratica* seedlings was worse (Figure 8). This is a more intuitive indication that the intraspecific competitive relationship constitutes a stress on the growth of *P. euphratica* seedlings, which is in line with most of the previous findings [28,48]. In addition, some studies have indicated that large plant species suffer greater stress and damage under intraspecific competition. Different plants have their suitable growth densities, which are closely related to environmental conditions, the plant’s own characteristics and interactions with other plants [50,51]. Therefore, understanding the intraspecific relationships and density-dependent regulation of *P. euphratica* under stress environments provides crucial guidance for optimizing planting density and improving resource use efficiency in arid zone vegetation restoration [43,49].

## 4. Materials and Methods

### 4.1. Study Sites and Materials

This research was conducted outdoors in the experimental field of the College of Ecology and Environment at Xinjiang University (87°73′ E, 43°84′ N). The field situated at an altitude of approximately 920 m, experiences a temperate continental arid climate, and characterized by an average annual temperature of 7.3 °C and an average annual precipitation of 227 mm.

The *Populus euphratica* seedlings were purchased in the Bayingolin Mongolian Autonomous Prefecture of Xinjiang, and none of the seedlings germinated in their initial state. We collected soil from the test field, removed impurities and mixed it with nutrient soil and vermiculite in the ratio of 6:3:1 for potting the test plants. We used white plastic pots with a height of 40 cm and an upper diameter of 30 cm. When planting, each pot should be filled with soil weighing approximately 15 ± 1 kg.

### 4.2. Experimental Design

The experiment began in late April with the transplantation of uniformly sized *P. euphratica* seedlings. These seedlings were randomly arranged in either single- or double-planted configurations. For the single-planted group, each seedling was positioned at the center of its pot. In the double-planted group, two seedlings were planted per pot with a separation of 7–10 cm. A few stones were placed at the bottom drainage hole, which served to ensure proper water drainage and prevent the loss of soil. After planting, the seedlings were shaded to ensure survival and watered every 5 days with 1 L per pot. After germination and full acclimatization, the seedlings were exposed to full light by removing shading. Thirty pots of single-planted and thirty pots of double-planted healthy *P. euphratica* seedlings were selected for stress treatment.

The stress treatments were administered once every seven days. This regimen began in July and continued for a total of 70 days. The experiment was conducted in a randomized block group design with five treatments: control (CK), low drought stress (D1), high drought stress (D2), low salt stress (S1), and high salt stress (S2). For the drought stress treatment, the irrigation volumes for CK, D1, and D2 were 1 L, 0.7 L, and 0.4 L per application, respectively. For the salt stress treatment, the CK, S1, and S2 groups were irrigated with 1 L of salt solution containing 0, 200, and 400 mmol L^−1^ NaCl per application, respectively.

### 4.3. Morphology and Biomass Determination

The height and basal diameter of each *P. euphratica* seedling were measured before and after the stress treatment, and the difference was calculated to obtain the growth in the stress period. At the end of the stress treatment, *P. euphratica* seedlings were cut from the base of the ground and divided into aboveground and belowground parts, and then the branches and leaves of the aboveground parts were collected separately. The collected branches, leaves and roots were placed in paper bags and dried at 70 °C for 48 h to constant weight, then weighed their dry weights as the biomass of each part. The root/shoot ratio (R/S ratio) was calculated as total root biomass/(leaf biomass + stem biomass). All plant samples were processed for subsequent experiments.

### 4.4. Measurement of Photosynthetic Physiology

At the end of the stress treatments, photosynthetic physiological parameters of plant leaves: net photosynthetic rate (P_n_) and stomatal conductance (g_s_) were measured using a LI-COR 6800 portable photosynthetic measurement system (LI-COR Co., Ltd., Lincoln, NE, USA ). The light intensity was set at 1500 μmol·m^−2^·s^−1^. The measurements were conducted on a clear day between 08:00 and 11:00 Beijing Time. In each pot of seedlings, three fully expanded leaves were randomly selected for measurement, and their average value was taken as the experimental data for recording.

### 4.5. Determination of Soluble Sugar and Starch

Soluble sugars and starch were determined by anthrone colorimetry, weighing 0.02 g of plant samples, extracting them with ethanol reagent, centrifuging them twice in a water bath and then colorimetrically determining the soluble sugar content using distilled water plus a color development solution as a blank. The residue was digested with perchloric acid to extract starch and then centrifuged. The absorbance of the resulting supernatant was measured spectrophotometrically to calculate the starch content. Detailed assay methods are provided by Song et al. [52].

### 4.6. Determination of Proline

Proline content was determined according to the method of Bates et al. [53]. The procedure involved extracting a 0.05 g plant sample in sulfosalicylic acid using a boiling water bath, with subsequent cooling and centrifugation. After mixing the supernatant with glacial acetic acid, ninhydrin, and toluene, the mixture was heated in a boiling water bath. After cooling, the absorbance was measured at 520 nm using a UV-1900i spectrophotometer (SHIMADZU CORP., Suzhou, China) with toluene as a blank, and the concentration was calculated.

### 4.7. Data Processing

We applied the Relative Interaction Index (RII) to quantify intraspecific interactions among *Populus euphratica* seedlings. RII is suitable for evaluating performance differentials between solitary and neighbored growth conditions, distinguishing facilitative from competitive relationships [7]. The formulae of calculating RII are as follows:

$$RII={(X}_{N}-X_{R})/(X_{N}+X_{R})$$

where X_neighbor_ (X_N_) is the trait value of the target plant in the presence of a neighbor and X_removal_ (X_R_) denotes the trait value of the target plant when grown alone. The value of RII fluctuates between 1 and −1, where a negative value indicates that the competitive interaction is dominant, a positive value indicates that the facilitative interaction is predominant, and a greater distance from the value of 0 indicates that the strength of the interaction is greater.

Principal component analysis (PCA) is a commonly used dimensionality reduction technique that can be used to analyze the relationships between variables in a multivariate data set [54]. To comprehensively elucidate intraspecific relationships and their response mechanisms to stress, a multi-step statistical analysis was performed. We applied PCA to reduce the dimensionality of eight variables—plant height increment, basal diameter increment, total biomass, net photosynthetic rate, stomatal conductance, and the contents of leaf soluble sugars, starch, and proline—measured in both single- and double-planted *P. euphratica* seedlings across five treatments (control and four stress conditions). The original data were first standardized and subjected to correlation matrix-based eigenvalue decomposition, from which the first three principal components explaining over 80% of the total variance were retained [55]. Principal component scores for each treatment and planting pattern were then derived and used to calculate the RII_inter_ through the formula RII_inter_ = (SN − SR)/(SN + SR), thereby quantifying the trends of intraspecific relationships based on integrated plant performance across stress gradients.

All data in this study were subjected to normality (Shapiro–Wilk test) and homogeneity of variance (Levene’s test) tests. Following confirmation of these assumptions, the data were analyzed using independent samples *t*-test, one-way ANOVA, or two-way ANOVA. For significant effects in ANOVA, post hoc comparisons were conducted. All analyses were performed at a significance level of *p* < 0.05, and data are presented as mean ± SE. All data were processed and analyzed using SPSS 27.0 and plotted using Origin 2021.

## 5. Conclusions

This study analyzes the performance of *Populus euphratica* seedlings in response to drought and salt stress, utilizing both traditional and comprehensive indicators to reveal the intraspecific relationships of these seedlings and their variations along stress gradients. The results indicate that drought and salt stress have a more significant impact on the growth of double-planted seedlings compared to singly planted ones, with both methods revealing competitive interactions under these stresses. Specifically, competition intensifies with increasing drought stress while maintaining similar intensity along the salinity gradient. These findings reflect the complex interrelationships among intraspecific dynamics, stress types, and plant responses. Framed within the refined Stress Gradient Hypothesis, our study reveals a pattern of intensified intraspecific competition in *P. euphratica* seedlings with increasing environmental stress. Guided by density-dependent regulation theory, we elucidate the practical implications of intraspecific relationships for vegetation restoration. This work deepens the understanding of plant–environment interactions and provides a theoretical foundation for biodiversity conservation and ecological restoration in arid regions.

## Figures and Tables

**Table 1:** One- and two-factor analysis of variance (ANOVA) of morphological, photosynthetic physiological, and chemical characteristics of *P. euphratica* seedlings under plant number (N), drought stress (D), and salt stress (S).

Parameters	*p* Value				
	*F_N_*	*F_D_*	*F_S_*	F_N*D_	F_N*S_
Height difference	**0.000**	**0.027**	**0.002**	0.072	0.192
Base diameter difference	0.156	0.155	**0.003**	0.435	0.714
Above-ground biomass	**0.000**	**0.011**	**0.000**	0.746	0.200
Below-ground biomass	**0.000**	**0.003**	**0.000**	0.725	0.179
Total biomass	**0.000**	**0.005**	**0.000**	0.945	0.183
R/S ratio	0.904	**0.034**	**0.002**	0.153	0.893
*P_n_*	**0.011**	0.060	**0.004**	0.144	0.290
*g_s_*	**0.020**	0.086	**0.021**	0.159	0.698
Leaf soluble sugar content	0.094	**0.000**	**0.000**	0.620	0.301
Branch soluble sugar content	0.554	0.059	0.091	0.742	0.505
Leaf starch content	**0.002**	**0.000**	**0.000**	**0.007**	0.832
Branch starch content	**0.009**	0.101	**0.000**	0.315	0.878
Leaf proline content	0.888	**0.008**	**0.000**	0.581	**0.004**
Branch proline content	0.524	**0.000**	**0.000**	**0.002**	0.815

*F_N_*, number effect; *F_D_*, drought effect; *F_S_*, salinity effect; F_N*D_, number × drought interaction effect; F_N*S_, number × salinity interaction effect. The bolded data in the table indicates *p* < 0.05.

## Data Availability

The raw data supporting the conclusions of this article will be made available by the authors on request.
